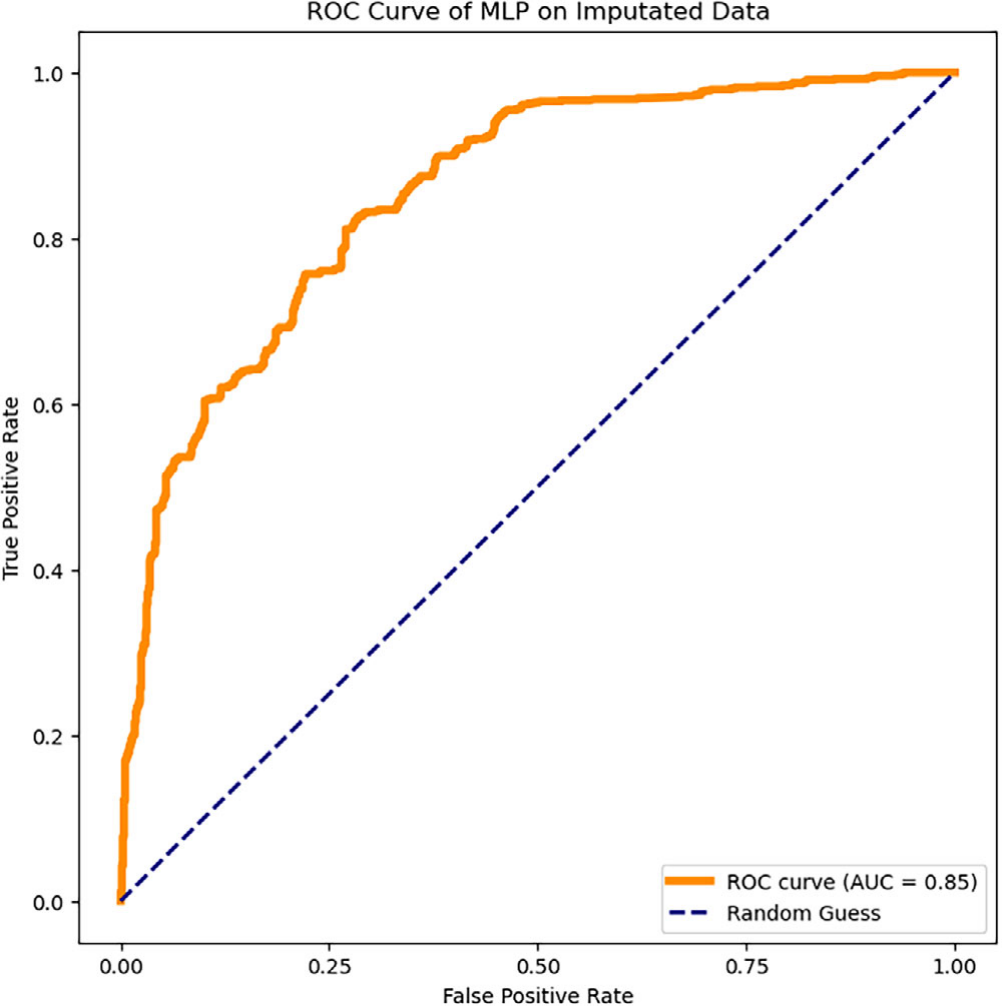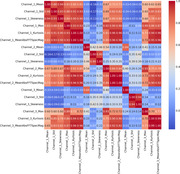# Detecting Major Depressive Disorder from EEG Signals as a Biomarker for Alzheimer’s Disease

**DOI:** 10.1002/alz.095081

**Published:** 2025-01-09

**Authors:** Sylvester O Orimaye, Jeremy Smith, Guy Colado, Sunvy Tong, Tomas Jager

**Affiliations:** ^1^ University of Health Sciences and Pharmacy, St. Louis, MO USA

## Abstract

**Background:**

Major Depressive Disorder (MDD) is a complex and multifaceted condition. Traditional screening methods may not capture the full spectrum of symptoms, causing delayed diagnoses or misdiagnoses. Further, MDD is highly prevalent among patients with mild cognitive impairment (MCI), which may then progress to Alzheimer’s disease (AD). Research suggests that MDD among patients with MCI substantially accelerates cognitive decline many years before the onset of AD. We show that a machine learning model can aid MDD diagnoses as a potential biomarker of AD.

**Method:**

Since MDD is associated with changes in prefrontal brain activity, we used electroencephalogram (EEG) signals from this brain region to train a two‐class multi‐layer perceptron (MLP) neural network model that discriminates between MDD patients and healthy controls. The MLP parameters include the lbfgs solver, three hidden layers, 1000 max iterations, alpha of 1e‐6, a relu function, tolerance level of 1e‐3, and a learning rate of 0.1. We used the Multi‐Modal Open Dataset for Mental Disorder Analysis (MODMA) data, which contains three EEG channels, including Fp1, Fp2, and Fpz locations. We extracted predictive features from each channel by transforming the raw EEG signals to train our model. Our features included the EEG signal’s maximum, mean, standard deviation, skewness, kurtosis, and mean absolute Fast Fourier Transform extended with multiple imputed data on the original 50 patients. This led to 1550 observations at 775 per MDD or control. Training and testing data was split using a ratio of 0.70 to 0.30, and experiments were performed using the Scikit‐learn Python library.

**Result:**

The experimental results indicate that the selected three‐channel EEG features can distinguish between MDD and healthy control subjects. The classification accuracy score was 76.8%, and the area under the curve (AUC) score was 85.3% (CI: 83.4% to 87.2%).

**Conclusion:**

We have demonstrated the effectiveness of using EEG signals to detect MDD in patients. The potential applications of our findings are the ability to aid in early medical diagnosis and serve as probable biomarkers of MCI and AD. Additional research is warranted to investigate more predictive features from the EEG signals.